# The association of early combined lactate and glucose levels with subsequent renal and liver dysfunction and hospital mortality in critically ill patients

**DOI:** 10.1186/s13054-017-1785-z

**Published:** 2017-08-21

**Authors:** Pedro Freire Jorge, Nienke Wieringa, Eva de Felice, Iwan C. C. van der Horst, Annemieke Oude Lansink, Maarten W. Nijsten

**Affiliations:** Department of Critical Care, University Medical Center Groningen, University of Groningen, PO Box 30001, 9700RB Groningen, The Netherlands

**Keywords:** Critical illness, Lactate, Glucose, Cori cycle, Acute kidney injury, Liver dysfunction, Organ failure, Mortality, Metabolism

## Abstract

**Background:**

The development of renal and liver dysfunction may be accompanied by initially subtle derangements in the gluconeogenetic function. Discrepantly low glucose levels combined with high lactate levels might indicate an impaired Cori cycle. Our objective was to examine the relation between early lactate and glucose levels with subsequent renal and liver dysfunction and hospital mortality in critically ill patients.

**Methods:**

Over a 4-year period (2011 to 2014), all adult patients admitted to our adult 48-bed teaching hospital intensive care unit (ICU) for at least 12 h were retrospectively analyzed. Lactate and glucose were regularly measured with point-of-care analyzers in all ICU patients. Lactate and glucose measurements were collected from 6 h before to 24 h after ICU admission. Patients with fewer than four lactate/glucose measurements were excluded. Patients received insulin according to a computer-guided control algorithm that aimed at a glucose level <8.0 mmol/L. Renal dysfunction was defined as the development of acute kidney injury (AKI) within 7 days, and liver function was based on the maximal bilirubin in the 7-day period following ICU admission. Mean lactate and mean glucose were classified into quintiles and univariate and multivariate analyses were related with renal and liver dysfunction and hospital mortality. Since glucose has a known U-shaped relation with outcome, we also accounted for this.

**Results:**

We analyzed 92,000 blood samples from 9074 patients (63% males) with a median age of 64 years and a hospital mortality of 11%. Both lactate quintiles (≤1.0; 1.0–1.3; 1.3–1.7; 1.7–2.3; >2.3 mmol/L) and glucose quintiles (≤7.0; 7.0–7.6; 7.6–8.2; 8.2–9.0; >9.0 mmol/L) were related with outcome in univariate analysis (*p* < 0.001). Acute Physiology and Chronic Health Evaluation (APACHE) IV, lactate, and glucose were associated with renal and liver dysfunction in multivariate analysis (*p* < 0.001), with a U-shaped relationship for glucose. The combination of the highest lactate quintile with the lowest glucose quintile was associated with the highest rates of renal dysfunction, liver dysfunction, and mortality (*p* < 0.001) with a significant interaction between lactate and glucose (*p* ≤ 0.001).

**Conclusions:**

Abnormal combined lactate and glucose measurements may provide an early indication of organ dysfunction. In critically ill patients a ‘normal’ glucose with an elevated lactate should not be considered desirable, as this combination is related with increased mortality.

**Electronic supplementary material:**

The online version of this article (doi:10.1186/s13054-017-1785-z) contains supplementary material, which is available to authorized users.

## Background

Derangements of lactate and glucose levels are common in critically ill patients [1–3]. Of the routinely available laboratory measurements, lactate has arguably the strongest relation with outcome in a broad variety of clinical settings [4] and, as a result, it is increasingly used to monitor the effectiveness of instituted therapy [5]. Since lactate and glucose monitoring has steadily gained popularity, optimal understanding of changes in glycometabolism may improve interpretation of acute clinical changes.

Lactate and glucose are linked through both glycolysis and gluconeogenesis, both pathways that are part of the Cori cycle. Gluconeogenesis which is performed by the liver and the kidney recycles circulating lactate into glucose [1, 6, 7]. Mild hypoglycemia can therefore be the result of dysfunction of the gluconeogenetic organs and it has been associated with impaired renal and liver function which affects outcome [8].

Most frequently, the stress reaction that accompanies acute critical illness directly induces both hyperlactatemia and hyperglycemia [9]. Thus hyperlactatemia without hyperglycemia might constitute already an abnormal response in the face of stress. Therefore we hypothesized that the combination of an elevated lactate with even a ‘normal’ glucose might be associated with an increased incidence of renal or liver dysfunction and hospital mortality.

## Methods

We conducted a retrospective study of all patients older than 18 years of age admitted to the intensive care unit (ICU) of the University Medical Center Groningen from 2011 to 2014. The anonymized data analysis in this study was performed in accordance with the guidelines outlined in Dutch legislation, and the study was approved by the medical ethics committee of our institution (Medisch Ethische Commissie, UMC Groningen, METc 2015/357). Because this was a retrospective study of routinely collected data, informed consent was not required by our ethics committee.

Anonymized patient information was obtained from the hospital’s electronic patient database. We recorded demographic data (age and gender), Acute Physiology and Chronic Health Evaluation (APACHE) IV score at admission, body weight, height, body mass index (BMI), presence of diabetes mellitus (DM), and steroid use within 18 h of ICU admission. We have implemented a computerized moderately tight glucose and potassium control protocol (GRIP) to keep glucose levels <8.0 mmol/L [10]. The amount of insulin administered during the first 24 h of ICU admission was recorded.

Blood gas analysis was performed by ABL 700 and 800 series analyzers, including lactate and glucose levels in arterial blood. These analyzers were present in the emergency department, operating room, and ICU. They were all regularly calibrated against the same standard.

In our analysis we included patients that had at least four glucose measurements and four lactate measurements between 6 h before and 24 h after admission to the ICU. Only patients who had an ICU stay of at least 12 h were included. For each patient, the mean lactate and mean glucose over the period of 6 h before to 24 h after admission to the ICU were subgrouped into quintiles.

Renal dysfunction was defined by the presence of acute kidney injury (AKI) according to the Kidney Disease Improving Global Outcomes (KDIGO) criteria in the 7-day period following ICU admission [11]. Liver dysfunction was defined by the maximal bilirubin in the 7-day period following ICU admission. The choice of bilirubin is based on bilirubin being the main marker for liver function in several organ failure scores, including the Sequential Organ Failure Assessment (SOFA) score [12].

Our main outcomes were the presence of renal dysfunction, liver dysfunction, and hospital mortality. We also recorded other markers of liver impairment such as the enzymes aspartate aminotransferase (AST), alanine aminotransferase (ALT), alkaline phosphatase (AP), and gamma-glutamyltransferase (GGT), as well as prothrombin time (PT).

The quintiles of the mean lactate, mean glucose and (glucose quintiles – mean glucose quintile)^2^ (to accommodate for a U-shaped relationship) were related to the presence of AKI, maximal bilirubin, and hospital mortality. Logistic regression was performed for the renal dysfunction and hospital mortality outcomes. Linear regression analysis was performed for liver dysfunction. We performed univariate and multivariate regression analyses for the three outcomes. In the multivariate analyses we included an interaction term between lactate and glucose (lactate quintile × glucose quintile). In order to test for possible influence of other covariates we performed multivariate regression analyses including the following covariates: APACHE IV score, steroid administration, DM, mean insulin dosage in the first 24 h of admission to the ICU, and glycemic variability (standard deviation of all glucoses obtained ≤24 h). These covariates were chosen based on their strong predictive value of mortality (APACHE IV) or due to their possible effect on glycometabolism (steroids, DM, and insulin). The multivariate regression analyses were performed with the Enter method. Normally distributed variables were compared using Student’s "*t* test" and non-normally distributed variables were compared using the Mann-Whitney "*U* test". Categorical variables were compared with the chi-square test. All analyses were performed using IBM SPSS® 23.

## Results

We analyzed a total number of 92,000 blood samples from 9074 patients admitted to the ICU between 2011 and 2014. The median (interquartile range (IQR)) age was 64 (54–72) and 63% were male. Of these patients, 1007 (11.1%) died during hospital stay.

Mean ± SD glucose levels were 8.1 ± 1.7 mmol/L and mean lactate levels were 1.8 ± 1.4 mmol/L over the whole patient population. Steroids were administered in 1212 (13%) patients and the mean insulin dose was 1.0 ± 1.3 IU/h. Frank hypoglycemia with a glucose level <2.2 mmol/L occurred in 0.6% of the patients, and with a glucose level <4.0 mmol/L in 4.3% of the patients (Table 2). The deceased patients were significantly older, of male gender, and had higher APACHE IV scores (Table 1; *p* < 0.001). Mean glucose and mean lactate were lower in survivors than in nonsurvivors (Table 2; *p* < 0.001). Both baseline and maximal liver enzymes and creatinine were lower in survivors than in nonsurvivors (Table 2; *p* < 0.001).

**Table 1:** Patient characteristics

Variable	All patients (*n* = 9074)	Hospital survivors (*n* = 8067)	Hospital nonsurvivors (*n* = 1007)	*p* value
Age, years	64 (53–72)	64 (53–72)	66 (56–75)	<0.001
Male	63% (5719)	63.4% (5118)	59.7% (601)	0.02
Admission through ED	27% (2415)	24.0% (1935)	47.7% (480)	<0.001
Type of admission				
Medical	7.0% (632)	6.7% (540)	9.1% (70)	0.006
Vascular, abdominal, miscellaneous surgery	20.9% (1900)	22% (1775)	12.4% (125)	<0.001
Neurosurgery	9.8% (893)	10.7% (866)	2.7% (27)	<0.001
Transplantation	1.2% (112)	1.3% (104)	0.8% (8)	NS
Cardiothoracic surgery	41.0% (3716)	43.9% (3542)	17.3% (174)	<0.001
Trauma	3.1% (280)	3.2% (258)	2.2% (22)	NS
Miscellaneous	17.0% (1541)	12.2% (982)	55.5% (559)	<0.001
APACHE IV score	48 (35–64)	45 (33–59)	87 (67–113)	<0.001
Body mass index, kg/m^2^	25.9 (23.5–28.7)	26.0 (23.7–28.8)	25.5 (23.1–28.2)	0.001
Diabetes	17% (1534)	16.6% (1337)	19.6% (197)	0.02
Mean 24 h insulin (IU/h)	0.63 (0.08–1.35)	0.62 (0.06–1.31)	0.77 (0.16–1.74)	<0.001
Steroids within 18 h of ICU admission	13% (1209)	12.0% (966)	24.1% (243)	<0.001

Values are presented as % (*n*) or medians (interquartile range) as appropriate

*APACHE* Acute Physiology and Chronic Health Evaluation, *ED* emergency department, *ICU* intensive care unit, *NS* not significant

**Table 2:** Laboratory and clinical outcomes

Variable	All patients (*n* = 9074)	Hospital survivors (*n* = 8067)	Hospital nonsurvivors (*n* = 1007)	*p* value
Number of combined lactate/glucose measurements per patient in first 24 h	10 (7–12)	10 (7–12)	11 (8–14)	<0.001
Mean lactate (mmol/l)	1.5 (1.1–2.9)	1.5 (1.1–2.0)	1.9 (1.3–3.2)	<0.001
Mean glucose (mmol/l)	7.9 (7.1–8.7)	7.8 (7.1–8.7)	8.2 (7.3–9.3)	<0.001
Hypoglycemia incidence (per patient)				
Any glucose <2.2 mmol/L	0.4% (34)	0.3% (21)	1.3% (13)	<0.001
Any glucose <3.3 mmol/L	1.8% (162)	1.4% (109)	5.3% (53)	<0.001
Any glucose <4.0 mmol/L	4.3% (390)	3.5% (285)	10.4% (105)	<0.001
Baseline creatinine (μmol/L)	81 (67–102)	80 (66–99)	95 (70–141)	<0.001
Maximal creatinine (μmol/L)	81 (65–109)	79 (64–103)	123 (80–219)	<0.001
AKI	20.4% (7224)	16.9% (6707)	51.3% (517)	<0.001
Stage 1	9.9% (894)	9.2% (740)	15.3% (154)	<0.001
Stage 2	3.5% (319)	2.8% (223)	9.5% (96)	<0.001
Stage 3	7.3% (664)	4.9% (397)	26.5% (267)	<0.001
Baseline bilirubin (μmol/L)	8 (6–14)	8 (5–13)	9 (6–18)	<0.001
Maximal bilirubin (μmol/L)	10 (7–15)	10 (7–15)	12 (8–24)	<0.001
Baseline ALT (IU/L)	25 (17–42)	25 (17–40)	31 (66–18)	<0.001
Maximal ALT (IU/L)	34 (20–70)	32 (19–64)	57 (27–177)	<0.001
Baseline AST (IU/L)	28 (22–45)	27 (21–42)	41 (25–92.5)	<0.001
Maximal AST (IU/L)	51 (30–101)	48 (30–89)	97.5 (44–323.5)	<0.001
Baseline GGT (IU/L)	37 (23–71)	36 (23–67)	49 (27–107)	<0.001
Maximal GGT (IU/L)	43 (21–106)	39 (20–98)	75 (36.5–152)	<0.001
Baseline AP (IU/L)	72 (57–93)	71 (57–91)	80 (58–119)	<0.001
Maximal AP (IU/L)	72 (54–108)	71 (53–104)	89 (63–141)	<0.001
Baseline PT (IU/L)	12.1 (11.3–14.1)	12.1 (11.2–14)	12.9 (11.5–15.6)	<0.001
Maximal PT (IU/L)	12.7 (11.9–14.2)	12.6 (11.8–14.0)	14.0 (12.1–18.6)	<0.001
ICU length of stay (days)	1.0 (0.9–3.5)	1.0 (0.9–2.8)	3.9 (1.6–9.4)	<0.001
Hospital length of stay (days)	11.1 (7.1–19.1)	11.2 (7.2–19.2)	7.3 (2.9–17.5)	<0.001

Values are presented as % (*n*) or medians (interquartile range) as appropriate

*AKI* acute kidney injury, *ALT* alanine aminotransferase, *AP* alkaline phosphatase, *AST* aspartate aminotransferase, *GGT* gamma-glutamyltransferase, *ICU* intensive care unit, *PT* prothrombin time

### Univariate associations of lactate and glucose with renal and liver dysfunction and hospital mortality

**Fig. 1:**
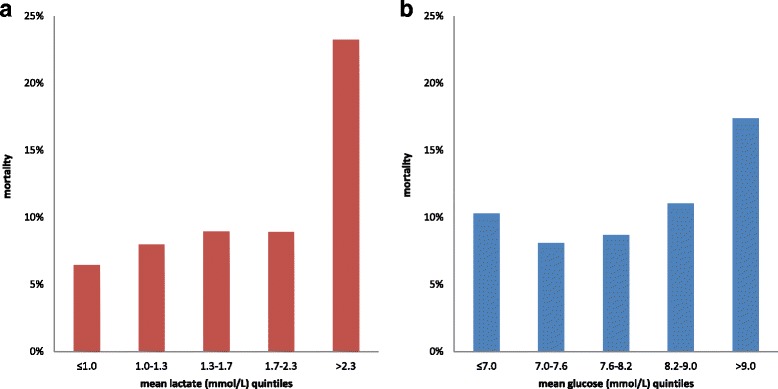
Univariate relationship between lactate and glucose and mortality. Lactate quintiles and hospital mortality (*left panel*) and glucose quintiles and hospital mortality (*right panel*). Note the U-shaped relationship for glucose. Both relationships deviate from the uniform distribution (*p* < 0.001)

Lactate had a positive relationship with mortality, with the last quintile having the highest hospital mortality rate (Fig. 1a; *p* < 0.001). Glucose showed a characteristic U-curve association with mortality (Fig. 1b; *p* < 0.001) with the first quintile having a higher hospital mortality rate than the second quintile.

In univariate regression analyses lactate quintiles, glucose quintiles, and (glucose quintile – mean glucose quintile)^2^ were positively associated with AKI and hospital mortality (Table 3; *p* < 0.001).

**Table 3:**
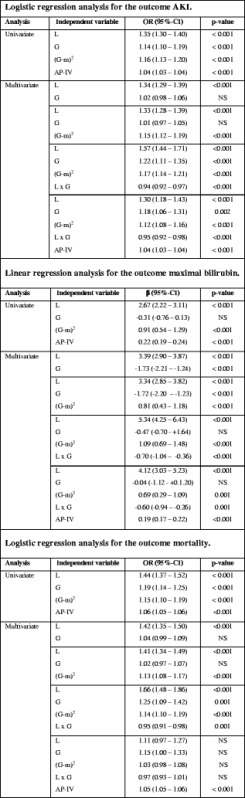
Regression analyses

*The statistical inference in the logistic regression is presented as an odds ratio (OR) (95% confidence interval (CI)) and in the linear regression as a β-coefficient (β) (95% CI)

Multivariate analyses for renal dysfunction (acute kidney injury (AKI); top third of table), liver dysfunction (maximal bilirubin; middle third of table), and mortality (bottom third of table) were performed with and without Acute Physiology and Chronic Health Evaluation (APACHE) IV (AP-IV) as an independent factor

Regression analyses are shown between lactate quintiles (L), glucose quintiles (G), (glucose quintile – mean)^2^ (G–m)^2^, and the interaction term (lactate quintile × glucose quintile (L × G)), and outcome

In order to accommodate for the known U-shaped relationship of glucose with outcome, the squared factor (glucose quintile – mean glucose quintile)^2^ is used. The term (lactate quintile × glucose quintile) was used to verify an interaction between lactate and glucose

Note the for both for AKI and mortality, the addition of the squared factor and of the interaction term improved the contribution of glucose in the multivariate model

*NS* not significant

### Multivariate associations of lactate and glucose with renal and liver dysfunction and hospital mortality

**Fig. 2:**
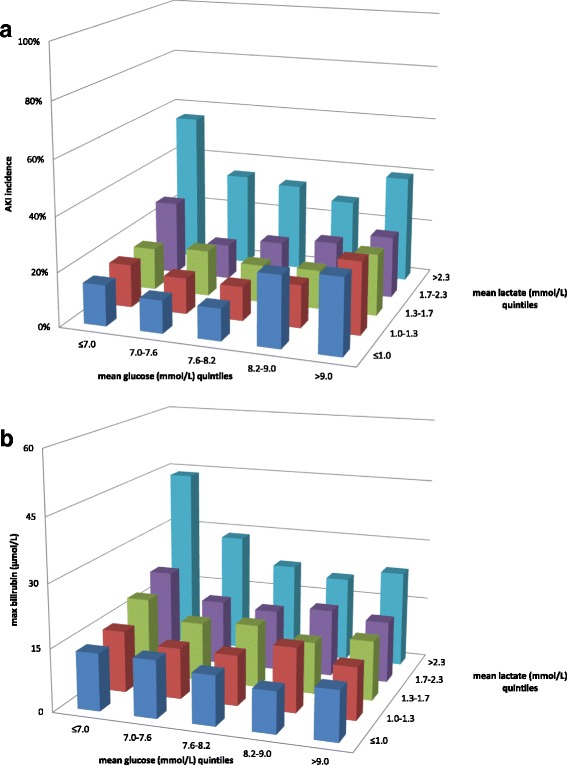
Multivariate relationship of lactate and glucose with renal and liver dysfunction. Combined early lactate and glucose and incidence of subsequent acute kidney injury (*AKI*; *upper panel*) and liver dysfunction as reflected by maximal bilirubin (*lower panel*). Note that the outcome is worst in the upper left corners, reflecting patients with the highest lactate and lowest glucose (*p* < 0.001)

The incidence of AKI was higher when a patient belonged in the lowest quintile of glucose level combined with elevated lactate level. The relationship of combined lactate and glucose with AKI is U-shaped with the incidence of AKI having a nadir in the mid-glycemic range and increasing towards the lower and higher glycemic quintiles in the presence of elevated lactate (Fig. 2a; *p* < 0.001).

The relation between the combination of lactate and glucose quintiles with bilirubin was one of increased bilirubin levels in the left upper quadrant of the plot (i.e., the mean bilirubin increases with increasing lactate and lower glucose) (Fig. 2b; *p* < 0.001).

In a multivariate logistic regression analysis the glycometabolic parameters, including the lactate × glucose interaction term, was significantly associated with AKI and bilirubin (Table 3). Introducing APACHE-IV score as a covariate did not significantly alter the relationship.

**Fig. 3:**
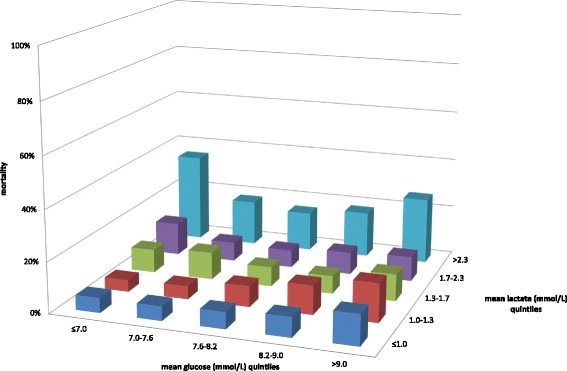
Lactate and glucose and mortality. Combined early lactate and glucose quintiles and hospital mortality. Note the sharply different mortality rates for the various combinations of lactate and glucose. The highest mortality rate is seen in patients in the upper left corner, i.e., with a mean lactate >2.3 mmol/L and a mean glucose ≤7.0 mmol/L (*p* < 0.001)

When lactate and glucose quintiles were plotted together against mortality we observed a U-shaped association between the glucose quintiles and mortality, mainly in the highest and in the second highest quintile of mean lactate (Fig. 3; *p* < 0.001).

**Fig. 4:**
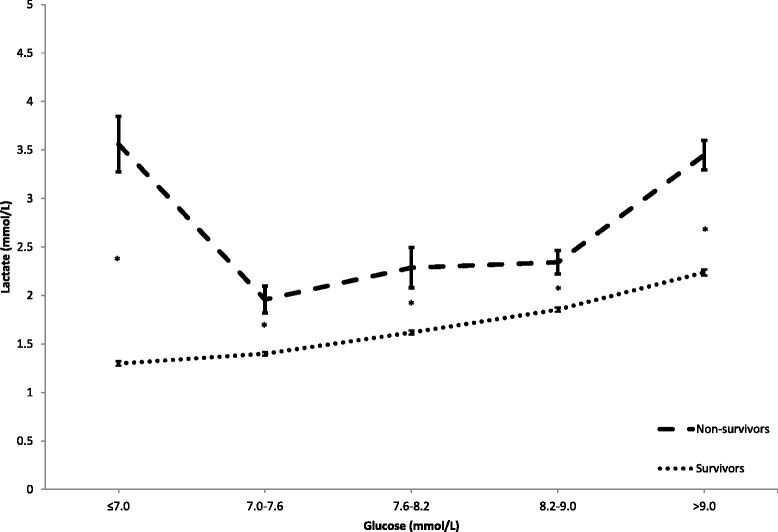
Mean lactate levels according to glucose quintile in survivors and nonsurvivors. Error bars represent the standard error of the mean. Note that in the lowest glucose quintile lactate levels are most discrepant between survivors and nonsurvivors. *p < 0.001 between survivors and nonsurvivors

The mean lactate versus the glucose quintiles displayed a positive linear relationship in the survivors, while nonsurvivors displayed a U-curve with increased mean lactate at both extremes of glycemia. Across all glycemic quintiles, nonsurvivors had significantly higher mean lactate (Fig. 4; *p* < 0.001).

In multivariate logistic regression the glycometabolic parameters, including the lactate × glucose interaction term, were significantly associated with hospital mortality (*p* ≤ 0.001), while, after inclusion of APACHE IV, only this factor was a significant predictor of mortality (Table 3; *p* < 0.001).

Multivariate regression models for all outcomes that include steroid administration, DM, insulin dosage, and glycemic variability as covariates are shown in Additional file 1: Tables S4, S5, and S6.

### Associations of glycometabolic parameters with other variables

Supplementary data are provided in Additional file 2: Figures S5–S16. For the combined glucose and lactate quintiles the number of patients (Additional file 2: Figure S5), APACHE IV (Additional file 2: Figure S6), presence of DM (Additional file 2: Figure S7), steroid administration (Additional file 2: Figure S8), insulin dose (Additional file 2: Figure S9), maximal PT (Additional file 2: Figure S10), and the liver enzymes AST (Additional file 2: Figure S11), ALT (Additional file 2: Figure S12), AP (Additional file 2: Figure S13), and GGT (Additional file 2: Figure S14) are shown. The glycemic variability for all glucose and lactate quintile combinations is shown in Additional file 2: Figure S15. Predicted mortality calculated from the model with only the glycometabolic variables (lactate quintiles, glucose quintiles, (glucose quintile – mean glucose quintile)^2^, and interaction term) is shown in Additional file 2: Figure S16.

## Discussion

In our study of a large heterogeneous cohort of ICU patients who received uniform glucose control, we found a strong relationship between lactate and glucose levels and outcome. Our hypothesis was that the combined assessment of early lactate and glucose would provide additional information on metabolic impairment and the associated risk for subsequent renal or liver dysfunction and mortality. A high lactate combined with a low glucose was associated with the highest risk of AKI, liver dysfunction, and hospital mortality. This finding is evident when combined lactate and glucose are graphically represented against these outcomes (Figs. 2 and 4). It is also reflected in the clear statistical interaction between lactate and glucose with respect to outcome in the multivariate analyses (Table 3). In agreement with many other studies, there was a strong association between elevated early lactate levels and mortality, and lactate levels were higher in nonsurvivors in all glucose quintiles, with disproportional increases in the lowest and highest glucose quintiles (Fig. 4). The utility of accommodating the U-shaped relation of glucose with outcome was also underscored by the multivariate analysis (Table 3).

Lactate and glucose production are strongly intertwined. In critically ill patients, the stress response is a common denominator for hyperlactatemia and hyperglycemia [9, 13, 14].

Adrenergic stimuli induce increased glycolysis leading to lactate production as well as glucose release from glycogen reserves [15]. We previously observed a parallel increase in lactate and glucose after the administration of pharmacological doses of corticosteroids [16]. In this study, we saw a comparable effect of steroids and glycemia on lactate levels (Additional file 1: Table S7).

Although it has long been known that the multifaceted acute stress response is a key determinant of hyperglycemia in the critically ill, the Leuven trial from 2001 suggested that decreasing glucose levels with insulin administration improved outcome [17]. Subsequent studies have called into question the wisdom of aggressive insulin therapy and also clearly demonstrated that intensive glucose control (target 4.5 to 6 mmol/L) is associated with more hypoglycemic events and increased mortality [18].

Both hyperglycemia and mild hypoglycemia are associated with worse outcome in critically ill patients [2, 19], but a recent large retrospective study found that in the hyperglycemic range the relation between glucose and outcome disappeared when lactate was taken into account [9]. Our study largely confirms this observation (Table 3). However, we believe that had they included the squared term to account for a U-shaped relationship of glucose and the lactate-glucose interaction term, the multivariate model could very much resemble the one found in our study.

The results of our study also demonstrate that the effect of the combination of lactate and glucose differs from the sum of the independent effects of glucose and lactate on outcomes, as indicated by the significant interaction terms (Table 3). Combined assessment of lactate and glucose apparently identifies different glycometabolic states. A state with both hyperlactatemia and hyperglycemia (i.e., the upper right corner in Figs. 2 and 3, and Additional file 2: Figures S5 to S16) could be interpreted as a stress response where the body still can generate ‘adaptive’ hyperglycemia [13]. In contrast, a state with elevated lactate without hyperglycemia (i.e., the upper left corner in the same figures) might be interpreted as state where the body is unable to develop hyperglycemia despite a stress response.

Since we observed a low incidence of frank hypoglycemia, glucose levels that would have been considered to be in the desired range (i.e., <7.0 mmol/l) could be considered as relative hypoglycemia in the context of elevated lactate levels. Our glucose control algorithm has a very low incidence of insulin-induced hypoglycemia [10] and prescribed very low insulin doses in the patients in the lowest glucose quintile (Additional file 2: Figure S9). We propose that the presence of stress lactatemia combined with ‘normoglycemia’ is a marker, not a mediator, of dysfunction of the only organs that can perform gluconeogenesis and participate in the Cori cycle—the liver and the kidneys.

The kidney is an important organ in the maintenance of glucose homeostasis [20]. The idea that the kidney plays a minor role in the overall gluconeogenesis compared to the liver is not correct as renal gluconeogenesis may account for up to 40% of overall gluconeogenesis in the postabsorptive state [21]. In patients with chronic renal failure, impaired gluconeogenetic capacity strongly increases the propensity for hypoglycemic events which is associated with increased risk of death in the context of sepsis or malnutrition [22]. Also, chronic renal failure has a negative impact on liver glycogenolysis and gluconeogenesis, thus further increasing these risks [23]. Lactate is the predominant precursor in epinephrine-stimulated gluconeogenesis of the kidney in normal healthy subjects who were infused with epinephrine to mimic physiological stress [6]. In our study, the patient group in both the highest mean lactate quintile and in the lowest mean glucose quintile had the highest incidence of AKI, suggesting that these glycometabolic parameters are associated, whether or not causally, to kidney function.

The liver is the most important organ that generates glucose through gluconeogenesis and glycogenolysis to maintain the normal glycemic range in the healthy fasting state. In the presence of stress stimuli, liver glycogen can be rapidly mobilized and the gluconeogenetic turnover increases [24]. The ability of the liver to perform gluconeogenesis requires large amounts of ATP generated by mitochondria. It has been estimated that up to 19% of all ATP used by the liver is used for gluconeogenesis in the physiological state [25]. This ATP turnover can increase up to 55% in sepsis [26] and the extra hepatic ATP production is used mainly to sustain the increased glucose output by gluconeogenesis [27]. Thus, in the setting of both depleted glycogen stores after 24 h of fasting and liver dysfunction, gluconeogenetic substrates such as lactate may increase while the appearance of blood glucose may be reduced [28].

Development of intraoperative hypoglycemia is highly predictive of posthepatectomy liver failure [29] and it is known that there is splanchnic net uptake of glucose and net release of lactate in acute liver failure [30]. Lactate levels have been shown to be significantly higher in patients with early hepatic dysfunction in the setting of acute circulatory failure [31] and the a priori presence of liver dysfunction as defined by previous medical history or initial laboratory measurements at presentation is significantly associated with impaired lactate normalization during resuscitation of severe sepsis and septic shock [32]. During acute liver failure, such as after paracetamol intoxication, gluconeogenesis can be extremely impaired with very elevated lactate levels (>20 mmol/L) and deep hypoglycemia (<1 mmol/L) [33]. Our study showed that patients with discrepantly elevated lactate and decreased glucose subsequently developed higher bilirubin levels and liver enzymes (Additional file 2: Figures S11 and S12) compared to patients with elevations of both lactate and glucose. This supports the notion that a combined assessment of glucose and lactate provides early additional information about the gluconeogenetic function of the liver during critical illness.

Our observations have specific practical consequences. First, with the expanding use of point-of-care blood gas analyses it is useful to interpret lactate and glucose in a combined fashion. In acutely ill patients who present with an increased lactate level, it has been well established that these patients, nearly irrespective of the underlying cause, are at increased risk of a poor outcome. When such patients present with normoglycemia or mild hypoglycemia as well they are at an even greater risk of poor outcome. Second, increased awareness of the heightened risk of frank hypoglycemia is warranted and may justify early prophylactic administration of intravenous glucose [34]. Also, the administration of glucose can support residual glycolytic metabolism of liver cells and reduce the risk of failure [35]. In the same way, it is known that preoperative increases in hepatic glycogen content through administration of insulin and glucose may have a protective effect against the development of postoperative liver failure [36, 37].

We believe our observations also have scientific implications. A prospective study evaluating the value of continuous lactate and glucose monitoring in at-risk patients might allow early identification of a divergence between lactate and glucose levels, and thus support tailored nutritional support with enteral feeding or glucose infusion [38]. At any rate, a better understanding of the bioenergetics of gluconeogenesis during critical illness is warranted and to what extent glucose administration may off-load the kidneys or the liver in patients with emerging organ failure.

This study has important limitations. As a retrospective and not an interventional study, direct causal relations cannot be proven, only made plausible. The key independent variables we used were the mean lactate and glucose within 24 h of ICU admission. We believed that the use of the means obtained over this relatively short period in a large number of patients was statistically the most straightforward choice. Our study focused only on the association between glycometabolism and dysfunction of gluconeogenetic organs. We did not have data on the function of other organ systems and therefore a possible association with such organ systems was not addressed and cannot be discarded at this point. Likewise, the observed associations do not provide proof of causal relations between lactate and glucose and organ dysfunction.

## Conclusions

In conclusion, the measurement of combined lactate/glucose levels at the point-of-care may provide a more complete picture of carbohydrate metabolism in critically ill patients. We found a strong relation between early hyperlactatemia with concomitant relative ‘normoglycemia’ and development of acute kidney injury and liver dysfunction.

## Additional files

Additional file 1: Table S4. Multivariate logistic regression analyses for the outcome AKI with other covariates. **Table S5.** Multivariate linear regression analyses for the outcome bilirubin with other covariates. **Table S6.** Multivariate logistic regression analyses for the outcome mortality with other covariates. **Table S7.** Linear regression analyses for the continuous outcome variable mean lactate with steroid administration and mean glucose as determinants. (PDF 290 kb)

Additional file 2: Figure S5. Number of patients per combined lactate and glucose quintiles. **Figure S6**. Mean APACHE IV score per combined lactate and glucose quintiles. **Figure S7**. Frequency of diabetes mellitus per combined lactate and glucose quintiles. **Figure S8**. Frequency of steroid administration per combined lactate and glucose quintiles. **Figure S9**. Mean insulin dose per combined lactate and glucose quintiles. **Figure S10**. Mean maximal PT per combined lactate and glucose quintiles. **Figure S11**. Mean maximal AST per combined lactate and glucose quintiles. **Figure S12**. Mean maximal ALT per combined lactate and glucose quintiles. **Figure S13**. Mean maximal AP per combined lactate and glucose quintiles. **Figure S14**. Mean maximal GGT per combined lactate and glucose quintiles. **Figure S15**. Glycemic variability in mmol/L during the first 24 h after ICU admission. **Figure S16**. Predicted mortality per combined lactate and glucose quintiles calculated from only glycometabolic parameters. (PDF 1.12 mb)

## Abbreviations

ALT: Alanine aminotransferase
AKI: Acute kidney injury
AP: Alkaline phosphatase
APACHE: Acute Physiology and Chronic Health Evaluation score
AST: Aspartate aminotransferase
BMI: Body mass index
DM: Diabetes mellitus
GGT: Gamma-glutamyltransferase
GRIP: Glucose regulation computer program for intensive care patients
ICU: Intensive care unit
IQR: Interquartile range
KDIGO: Kidney Disease Improving Global Outcomes
PT: Prothrombin time
