# Supplementary material for: The association of early combined lactate and glucose levels with subsequent renal and liver dysfunction and hospital mortality in critically ill patients
Source: Crit Care. 2017 Aug 21;21:218. doi: 10.1186/s13054-017-1785-z (PMC5563890; doi:10.1186/s13054-017-1785-z)
Supplement: Supplementary file 1 — Multivariate logistic regression analyses for the outcome AKI with other covariates. Table S5. Multivariate linear regression analyses for the outcome bilirubin with other covariates. Table S6. Multivariate logistic regression analyses for the outcome mortality with other covariates. Table S7. Linear regression analyses for the continuous outcome variable mean lactate with steroid administration and mean glucose as determinants. (PDF 290 kb) [file 13054_2017_1785_MOESM1_ESM.pdf]

ADDITIONAL FILE 1 accompanying "The association of early combined lactate and glucose levels with subsequent renal and liver dysfunction and hospital mortality in critically ill patients" by Freire Jorge et al.

This file contains supplementary tables 4, 5, 6 and 7.

Supplementary table 4

Multivariate logistic regression analyses for the outcome AKI with other co-variates.

Independent variable	OR (95%-CI)	p-value
L	1.57 (1.43 – 1.70)	< 0.001
G	1.16 (1.05 – 1.29)	0.003
(G-m)2	1.16 (1.13 – 1.20)	< 0.001
L x G	0.95 (0.92 – 0.98)	<0.001
DM	1.43 (1.25 – 1.64)	< 0.001
L	1.50 (1.38 – 1.64)	< 0.001
G	1.18 (1.07 – 1.30)	0.001
(G-m)2	1.16 (1.12 – 1.20)	< 0.001
L x G	0.95 (0.93 – 0.98)	<0.001
Steroids	2.51 (2.19 – 2.86)	< 0.001
L	1.60 (1.46 – 1.74)	< 0.001
G	1.12 (1.02 – 1.21)	0.029
(G-m)2	1.14 (1.10 – 1.18)	< 0.001
L x G	0.93 (0.91 – 0.96)	<0.001
Insulin	1.27 (1.21 – 1.33)	< 0.001
L	1.54 (1.41 – 1.68)	< 0.001
G	1.08 (0.98 – 1.20)	NS
(G-m)2	1.12 (1.09 – 1.16)	< 0.001
L x G	0.95 (0.92 – 0.97)	<0.001
G var	1.29 (1.23 – 1.36)	<0.001

The statistical inference in the logistic regression is presented as an odds ratio (OR) (95% - confidence interval) and in the linear regression as a β-coefficient (β) (95%-confidence interval).

L= lactate quintiles, G= glucose quintiles, $(G\text{-}m)^2$= (glucose quintile – mean quintile) squared, L x G= interaction term between lactate quintile and glucose quintile, AP-IV= APACHE-IV, DM = diabetes mellitus, G var = glycemic variability.

Supplementary table 5

Multivariate linear regression analyses for the outcome bilirubin with other co-variates.

Independent variable	β (95%-CI)	p-value
L	5.31 (4.23 – 6.40)	<0.001
G	-0.57 (-0.62 - +0.1.76)	NS
(G-m)2	1.08 (0.68 – 1.48)	0.001
L x G	-0.70 (-1.04 – -0.36)	0.001
DM	-0.72 (-2.45 – +1.00)	0.412
L	5.03 (3.95 – 6.12)	<0.001
G	0.18 (-0.99 - +1.34)	NS
(G-m)2	0.97 (0.58 – 1.37)	0.001
L x G	-0.65 (-0.99 – -0.31)	0.001
Steroids	7.28 (5.42 – 9.14)	<0.001
L	5.38 (4.29 – 6.46)	<0.001
G	0.27 (-0.92 - +1.46)	NS
(G-m)2	1.03 (0.63 – 1.43)	0.001
L x G	-0.73 (-1.07 – -0.39)	0.001
Insulin	0.55 (-0.06 – +1.16)	0.079
L	5.38 (4.29 – 6.46)	< 0.001
G	0.69 (-0.51 - +1.89)	NS
(G-m)2	1.16 (0.76 – 1.57)	< 0.001
L x G	-0.70 (-1.05 – -0.36)	<0.001
G var	-0.49 (-1.10 – +0.12)	NS

The statistical inference in the logistic regression is presented as an odds ratio (OR) (95% - confidence interval) and in the linear regression as a β-coefficient (β) (95%-confidence interval).

L= lactate quintiles, G= glucose quintiles, $(G\text{-}m)^2$= (glucose quintile – mean quintile) squared, L x G= interaction term between lactate quintile and glucose quintile, AP-IV= APACHE-IV, DM = diabetes mellitus, G var = glycemic variability.

Supplementary table 6 Multivariate logistic regression analyses for the outcome mortality with other co-variates.

Independent variable	OR (95%-CI)	p-value
L	1.66 (1.48 – 1.86)	<0.001
G	1.24 (1.09 – 1.42)	0.002
(G-m)2	1.14 (1.10 – 1.19)	<0.001
L x G	0.95 (0.91 – 0.98)	0.002
DM	1.03 (0.86 – 1.23)	NS
L	1.60 (1.42 – 1.79)	<0.001
G	1.21 (1.06 – 1.38)	0.004
(G-m)2	1.13 (1.09 – 1.18)	<0.001
L x G	0.95 (0.92 – 0.98)	0.004
Steroids	1.91 (1.62 – 2.26)	< 0.001
L	1.67 (1.49 – 1.87)	<0.001
G	1.21 (1.06 – 1.38)	0.005
(G-m)2	1.13 (1.09 – 1.18)	<0.001
L x G	0.94 (0.91 – 0.97)	0.001
Insulin	1.09 (1.03 – 1.15)	0.002
L	1.63 (1.46 – 1.83)	< 0.001
G	1.14 (1.00 – 1.31)	NS
(G-m)2	1.11 (1.06 – 1.16)	< 0.001
L x G	0.95 (0.91 – 0.98)	0.002
G var	1.19 (1.12 – 1.25)	< 0.001

The statistical inference in the logistic regression is presented as an odds ratio (OR) (95% - confidence interval) and in the linear regression as a β-coefficient (β) (95%-confidence interval).

L= lactate quintiles, G= glucose quintiles, $(G\text{-}m)^2$= (glucose quintile – mean quintile) squared, L x G= interaction term between lactate quintile and glucose quintile, AP-IV= APACHE-IV, DM = diabetes mellitus, G var = glycemic variability.

Supplementary table 7 Linear regression analyses for the continuous outcome variable mean lactate with steroid administration and mean glucose as determinants.

**Dependent variable**	**Analysis**	**Independent variable**	**β (95%-CI)**	**p-value**
Mean lactate	Univariate	Steroids	0.65 (0.56 – 0.73)	< 0.001
		Mean glucose	0.18 (0.17 – 0.20)	<0.001
	Multivariate	Steroids	0.57 (0.49 – 0.64)	< 0.001
		Mean glucose	0.17 (0.16 – 0.19)	< 0.001
