# Supplementary material for: The association of early combined lactate and glucose levels with subsequent renal and liver dysfunction and hospital mortality in critically ill patients
Source: Crit Care. 2017 Aug 21;21:218. doi: 10.1186/s13054-017-1785-z (PMC5563890; doi:10.1186/s13054-017-1785-z)
Supplement: Supplementary file 2 — Number of patients per combined lactate and glucose quintiles. Figure S6. Mean APACHE IV score per combined lactate and glucose quintiles. Figure S7. Frequency of diabetes mellitus per combined lactate and glucose quintiles. Figure S8. Frequency of steroid administration per combined lactate and glucose quintiles. Figure S9. Mean insulin dose per combined lactate and glucose quintiles. Figure S10. Mean maximal PT per combined lactate and glucose quintiles. Figure S11. Mean maximal AST per combined lactate and glucose quintiles. Figure S12. Mean maximal ALT per combined lactate and glucose quintiles. Figure S13. Mean maximal AP per combined lactate and glucose quintiles. Figure S14. Mean maximal GGT per combined lactate and glucose quintiles. Figure S15. Glycemic variability in mmol/L during the first 24 h after ICU admission. Figure S16. Predicted mortality per combined lactate and glucose quintiles calculated from only glycometabolic parameters. (PDF 1.12 mb) [file 13054_2017_1785_MOESM2_ESM.pdf]

Additional file 2

accompanying "The association of early combined lactate and glucose levels with subsequent renal and liver dysfunction and hospital mortality in critically ill patients" by Freire Jorge et al.

Contents:

For lactate and glucose quintiles:

number of patients	sFig5
APACHE-IV	sFig6
DM incidence	sFig7
steroid administration	sFig8
insulin dose	sFig9
max PT	sFig10
max AST	sFig11
max ALT	sFig12
max AP	sFig13
max GGT	sFig14
glycemic variability	sFig15
predicted mortality	sFig16

Suppl. figure 5 – Number of patients

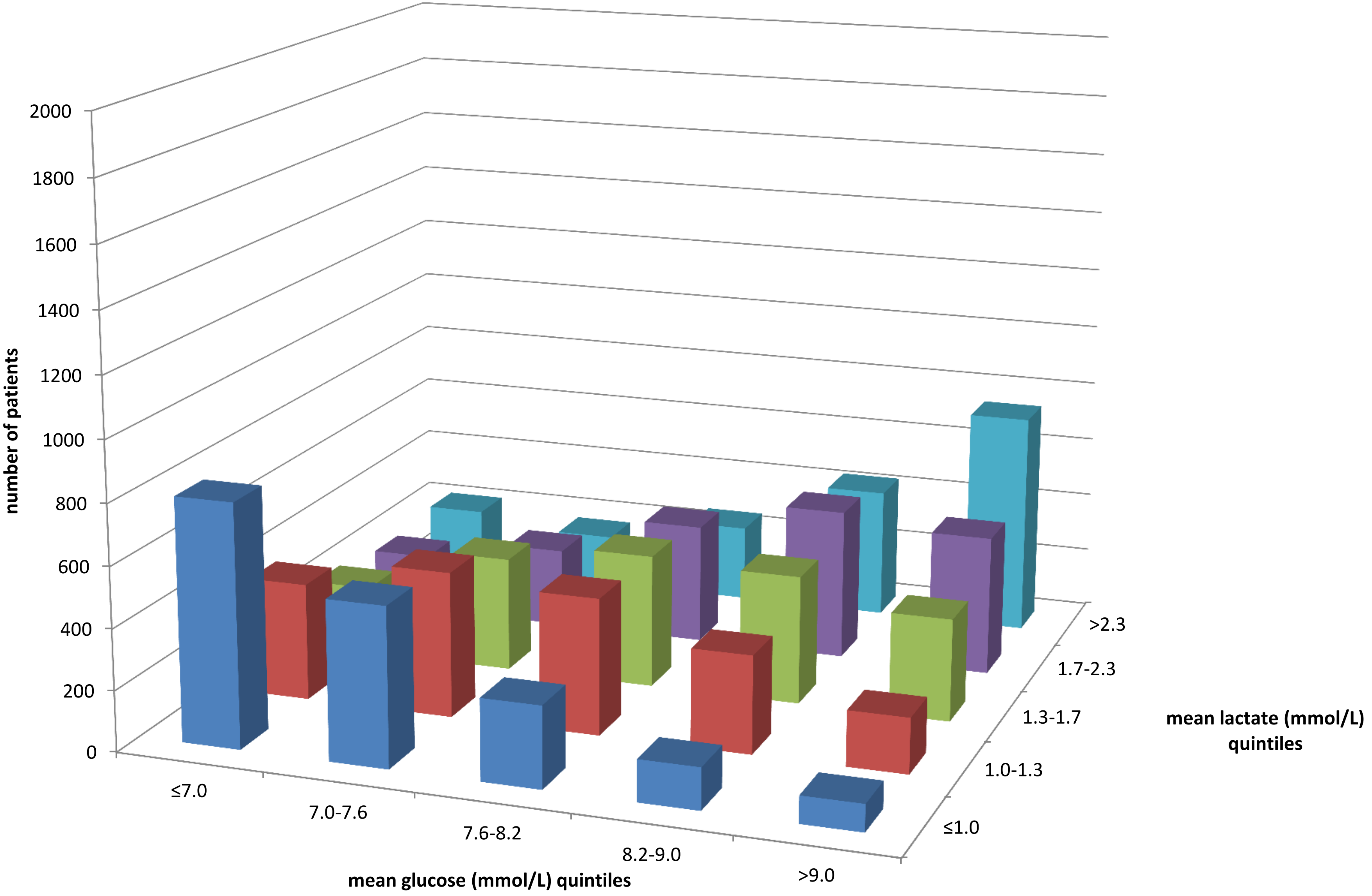

Legend for supplementary figure 5.

Number of patients for combined lactate and glucose quintiles.

Note that the majority of patients are in the diagonal between left-bottom to right-upper corner of the graph. The left-upper corner of the graph with lowest mean glucose and highest mean lactate represents 6% of our patient population.

Suppl. figure 6 – APACHE IV score

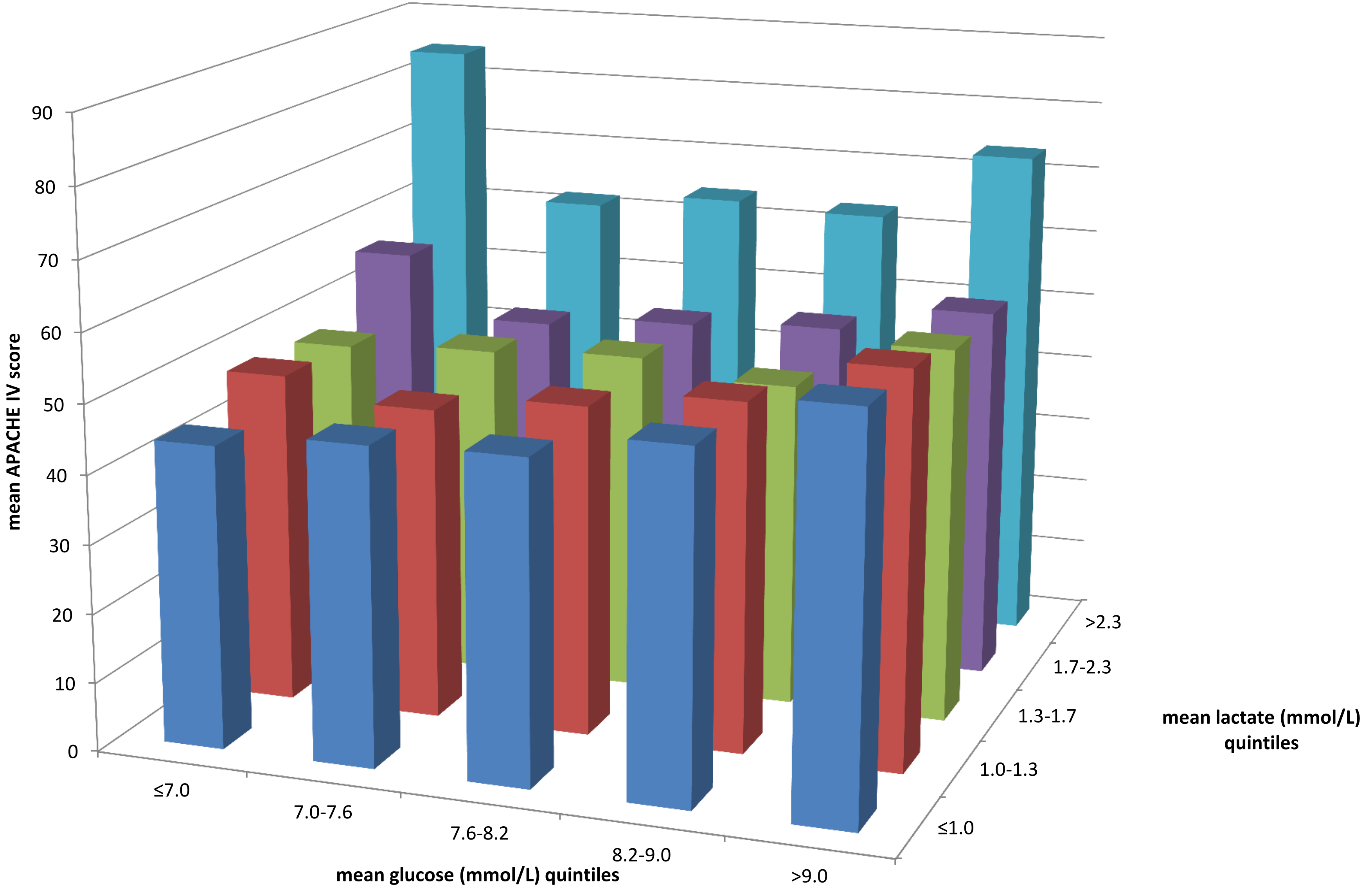

Legend for supplementary figure 6.

Mean APACHE-IV score for combined lactate and glucose quintiles.

Note that the highest quintile of lactate has overall the highest mean APACHE-IV score. Note also the U-curve that is observed in the highest lactate quintile across the glucose range. The group with the lowest glucose and highest lactate has the highest mean APACHE-IV score of all groups.

Suppl. figure 7 – Diabetes mellitus incidence (%)

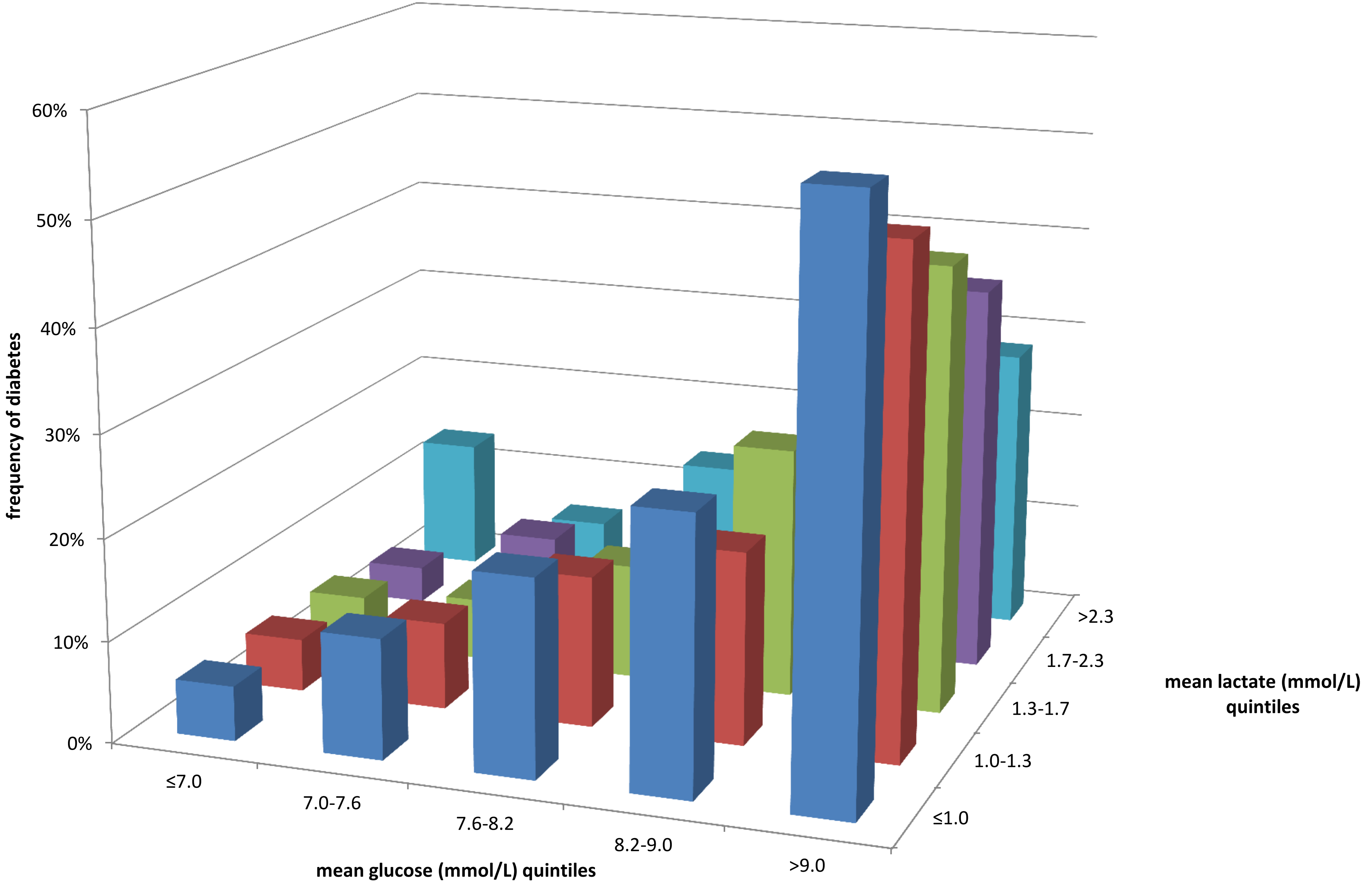

Legend for supplementary figure 7.

Frequency of Diabetes mellitus for combined lactate and glucose quintiles.

Note that the frequency of diabetes is the highest in highest glucose quintile. Also noticeable is a higher frequency of diabetes mellitus in the combined lowest glucose and highest lactate in comparison to the frequency observed in the second glucose quintile when combined with the highest lactate quintile.

Suppl. figure 8 – Steroid administration (%)

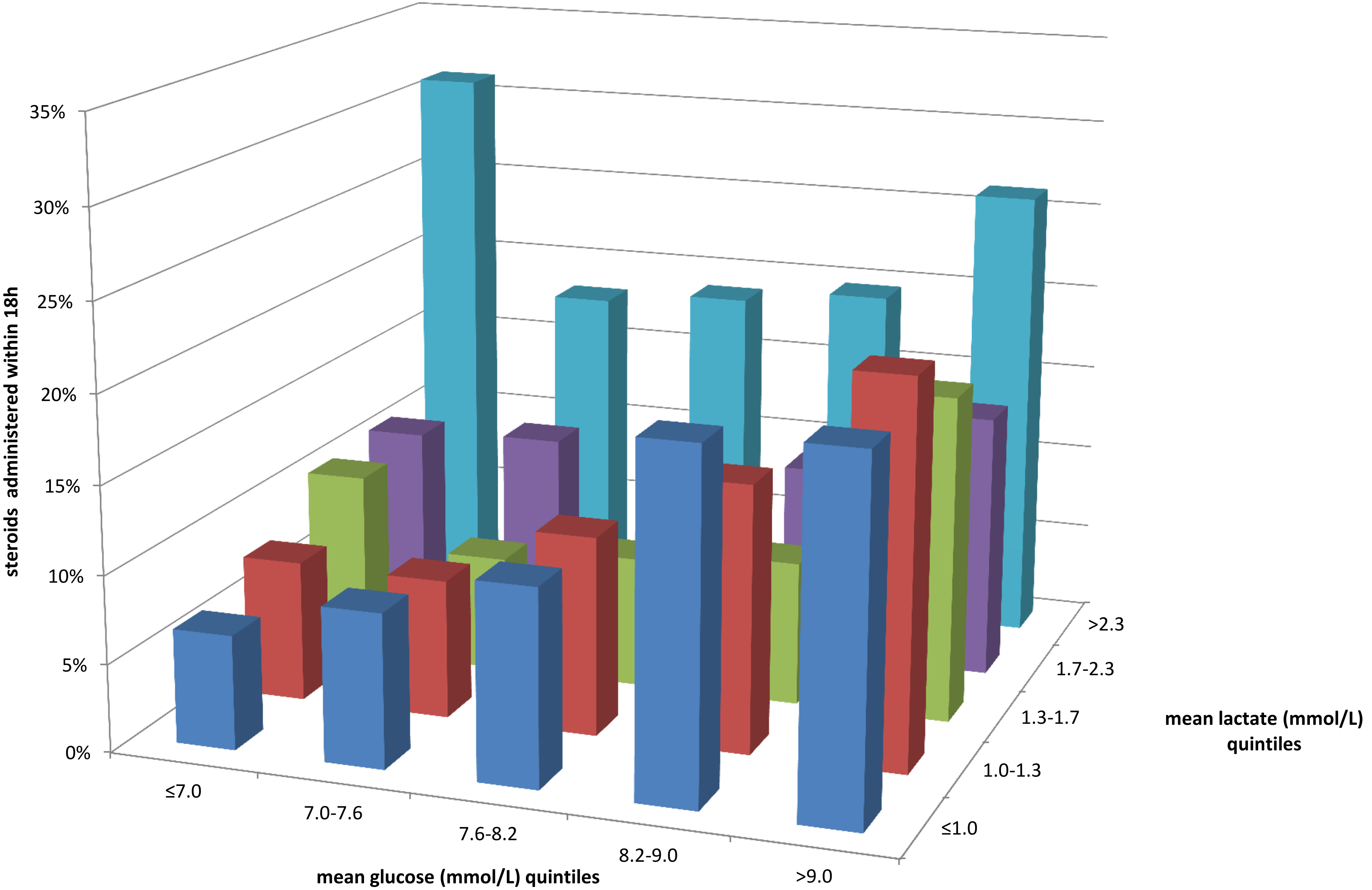

Legend for supplementary figure 8.

Frequency of steroid administration for combined lactate and glucose quintiles.

Note the U shape across the glycemic range for the highest lactate quintile. Also, the combined lowest glucose with the highest lactate quintiles has the highest frequency of steroid administration.

Suppl. figure 9 – Mean insulin dose (IU/h)

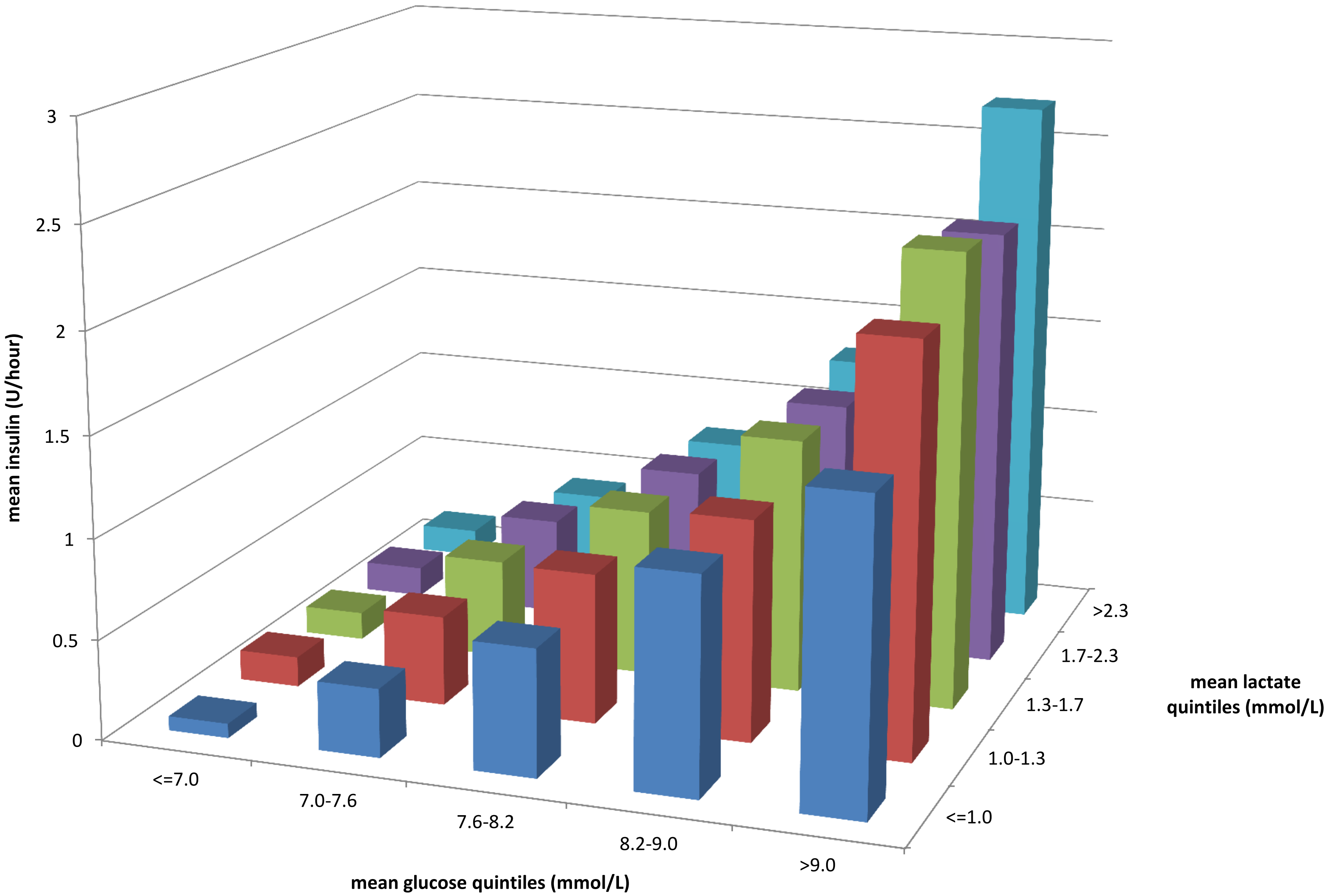

Legend for supplementary figure 9.

Mean insulin dose (IU/h) for combined lactate and glucose quintiles.

Mean insulin dose in IU/h during the first 24 hours after admission to the ICU for combined lactate and glucose quintiles. Note the characteristic increase in insulin dose with increasing glucose quintiles.

Suppl. figure 10 – Maximal prothrombin time (PT)

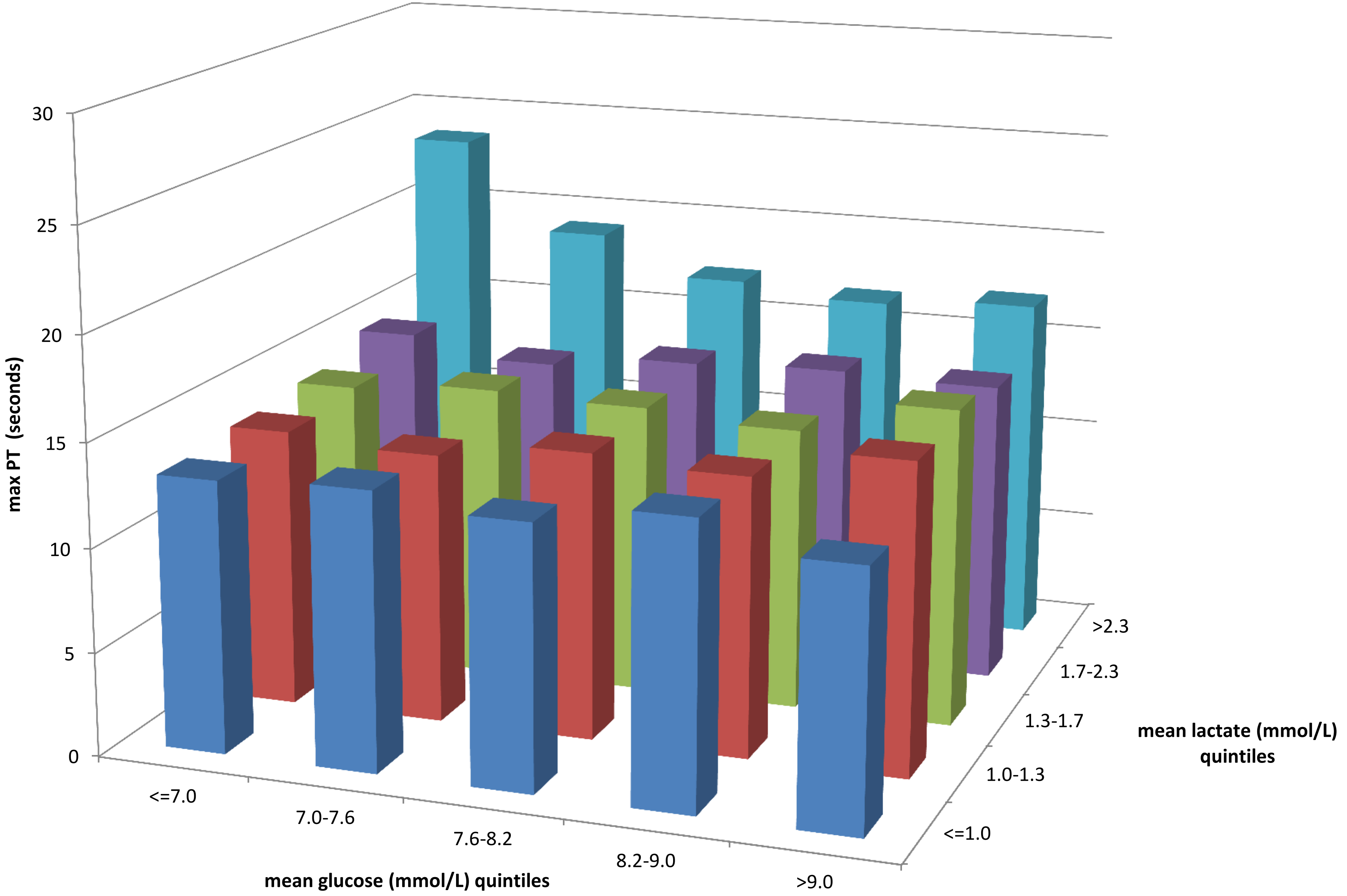

Legend for supplementary figure 10.

Mean maximal PT for combined lactate and glucose quintiles.

Note the increase in PT in the left-upper corner corresponding with impaired liver function in these patients.

Suppl. figure 11 – Maximal aspartate transaminase (AST; IU/L)

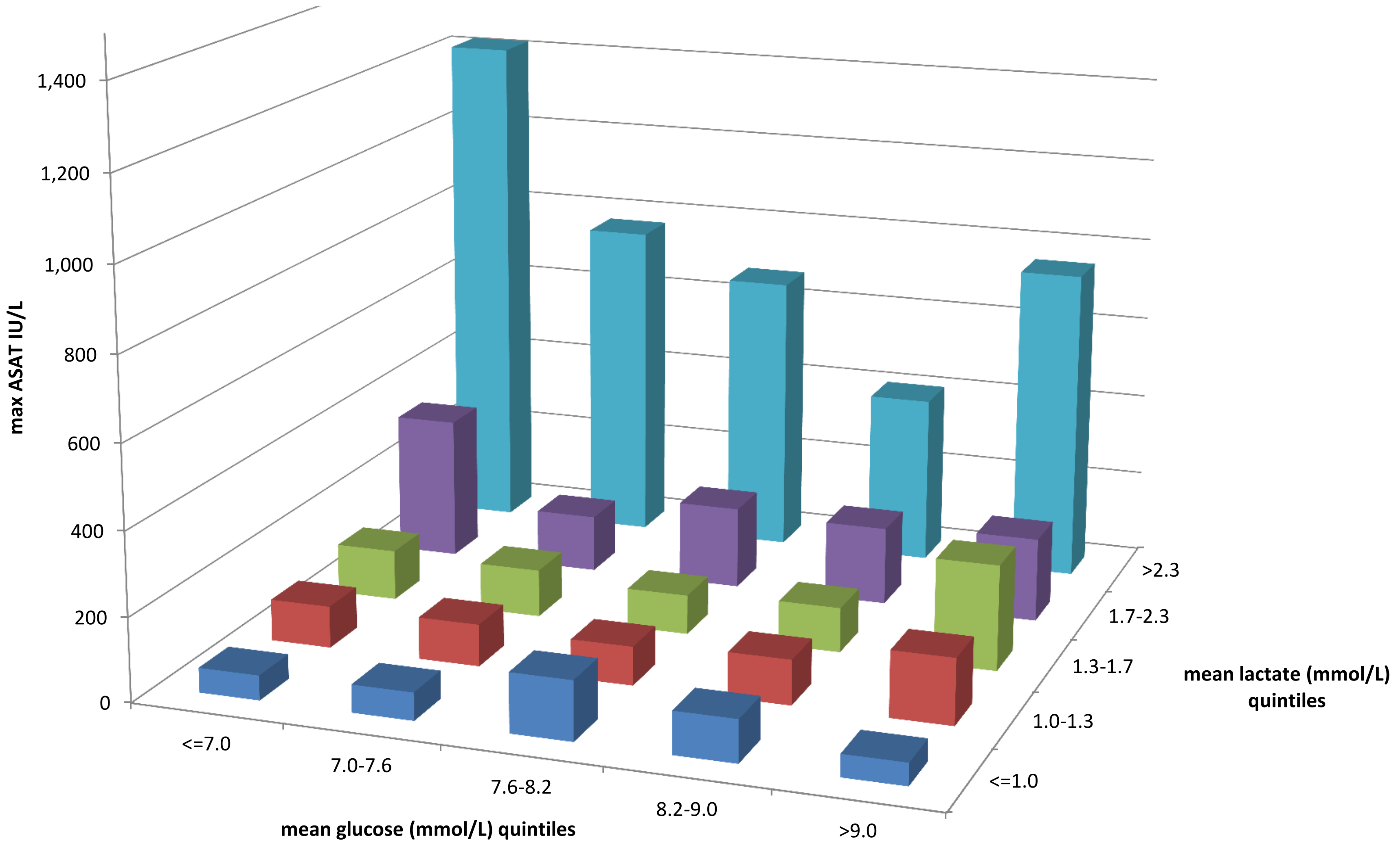

Legend for supplementary figure 11.

Mean maximal AST for combined lactate and glucose quintiles.

Note the highest value of AST is found in the left-upper corner of the graph, pointing to liver damage in these patients.

Suppl. figure 12 – Maximal alanine transaminase (ALT; IU/L)

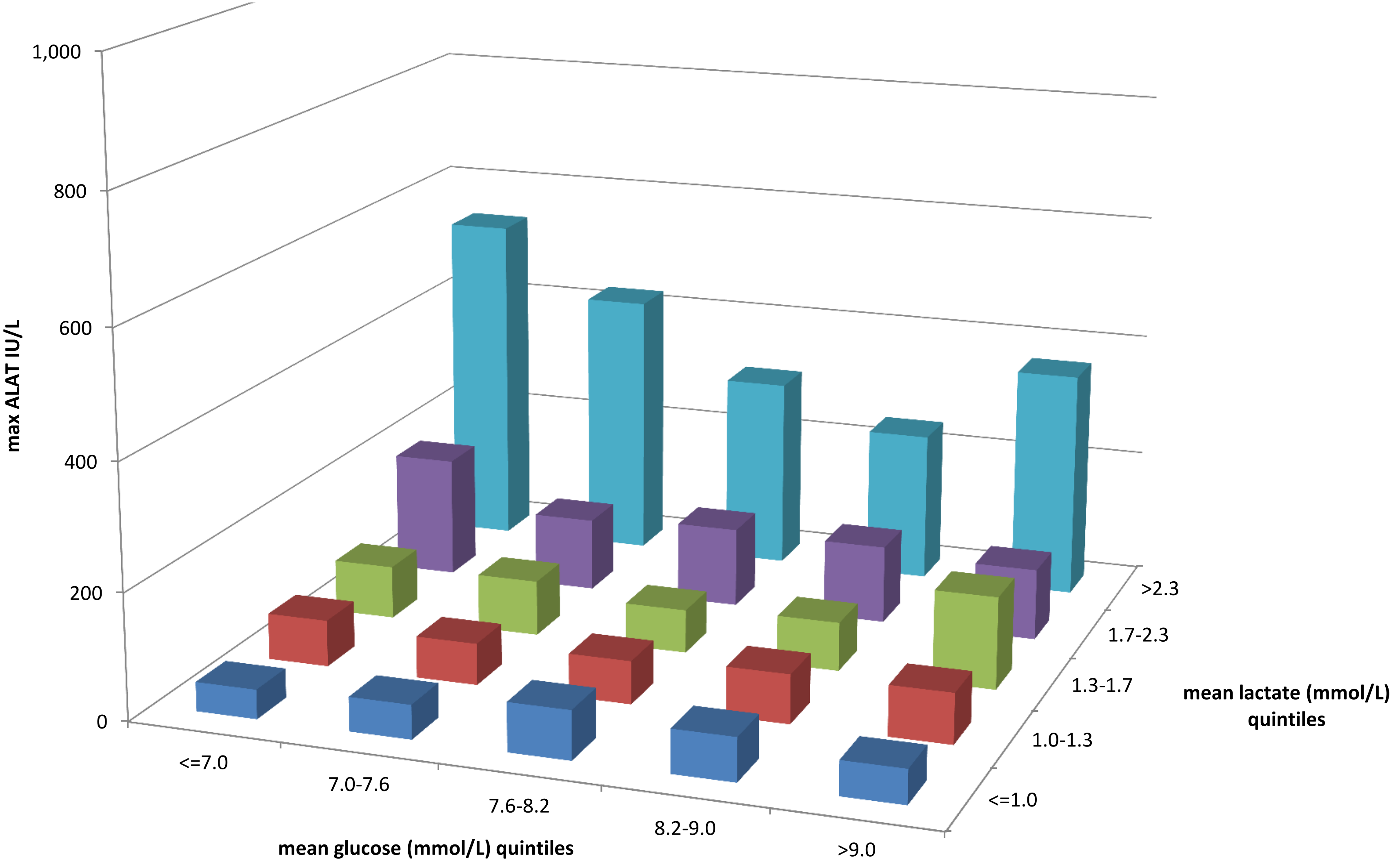

Legend for supplementary figure 12.

Mean maximal ALT for combined lactate and glucose quintiles.

Note the highest value of ALT is found in the left-upper corner of the graph, pointing to liver damage in these patients.

Suppl. figure 13 – Maximal alkaline phosphatase (AP; IU/L)

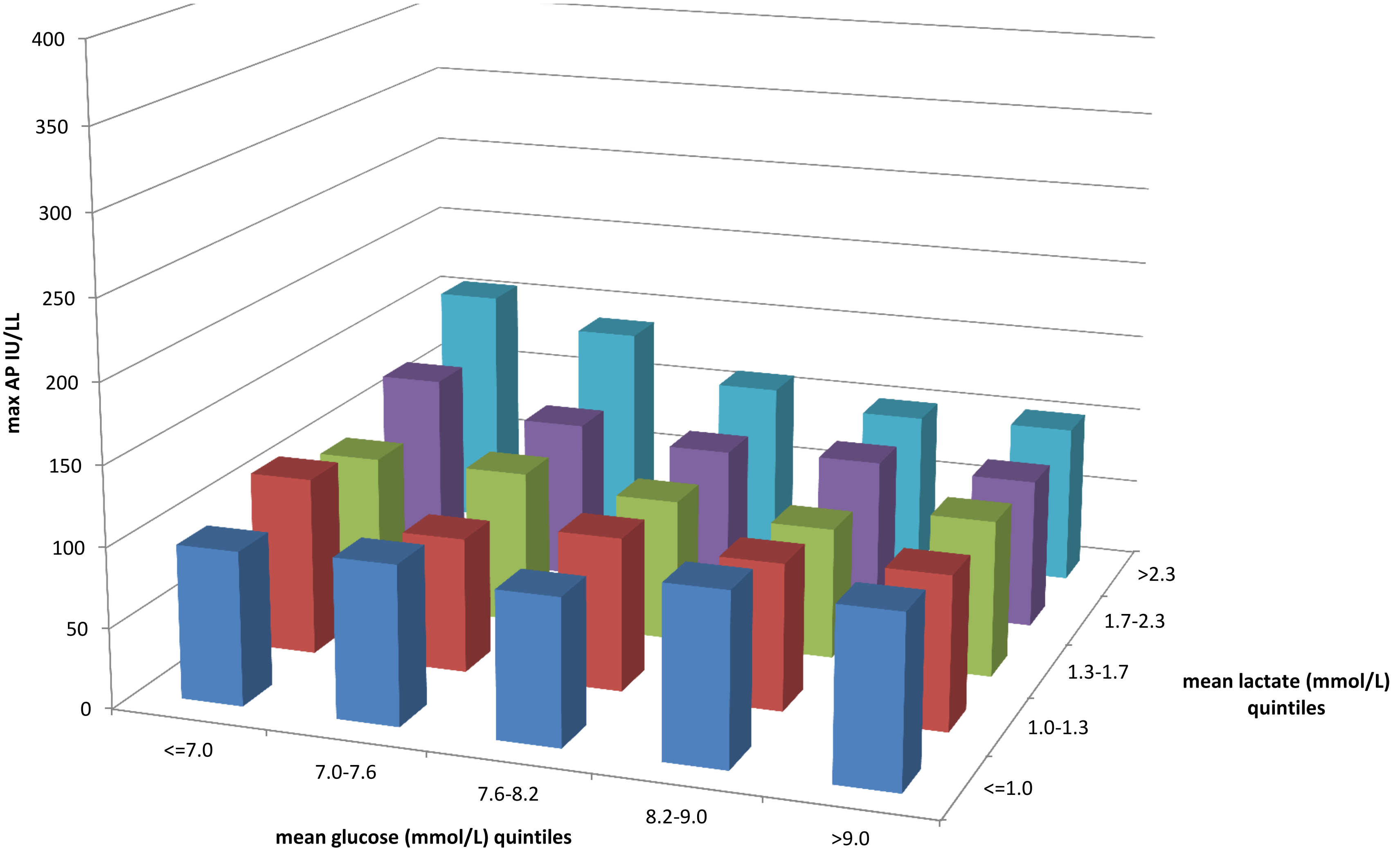

Legend for supplementary figure 13.

Mean maximal AP for combined lactate and glucose quintiles.

The value of AP increases lightly towards the left-upper corner of the graph.

Suppl. figure 14 – Maximal gamma-glutamyl transpeptidase (GGT; IU/L)

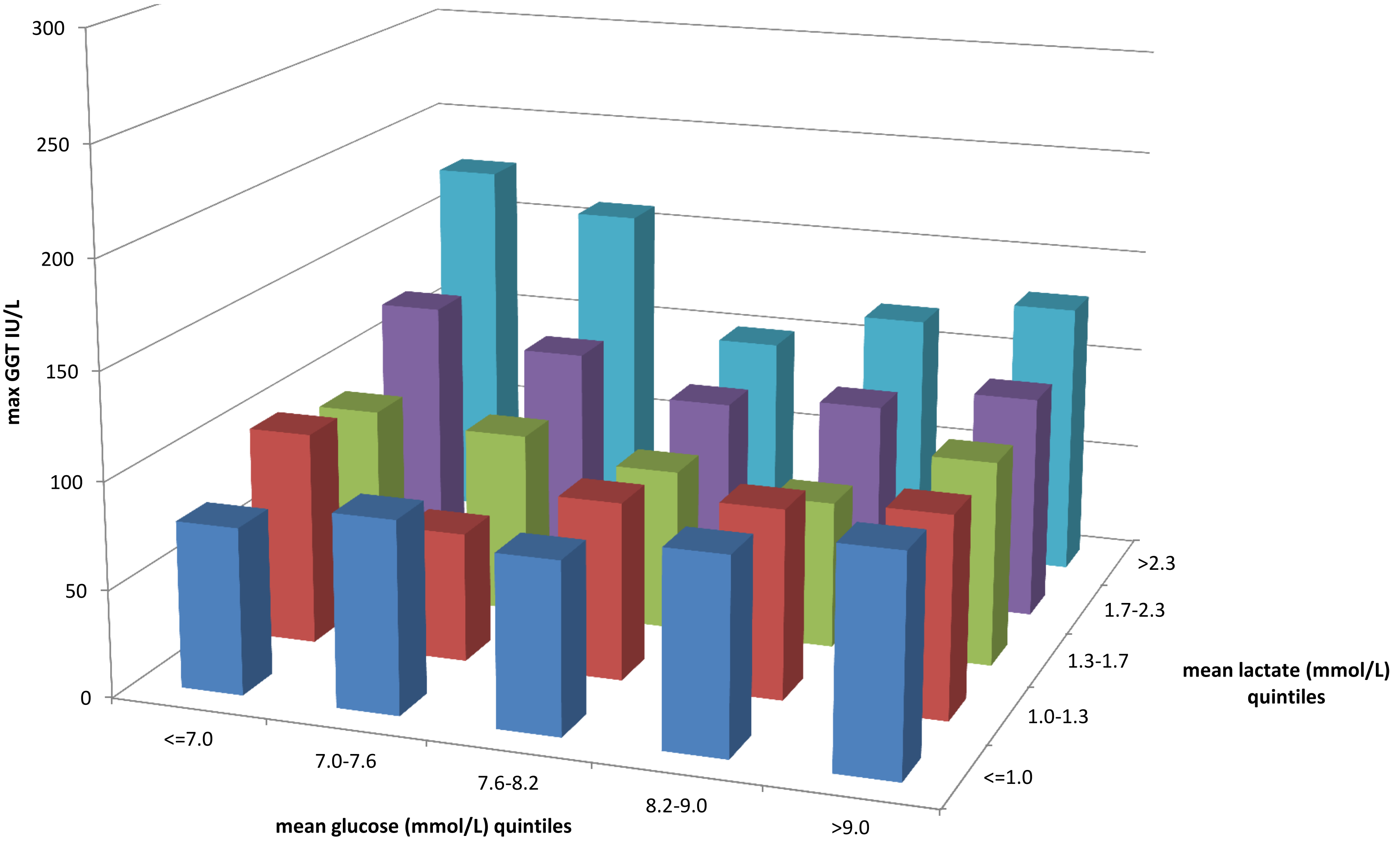

Legend for supplementary figure 14.

Mean maximal GGT for combined lactate and glucose quintiles.

The value of GGT increases towards the left-upper corner of the graph, suggesting liver impairment in these patients.

Suppl. figure 15 – Glycemic variability (SD; mmol/L)

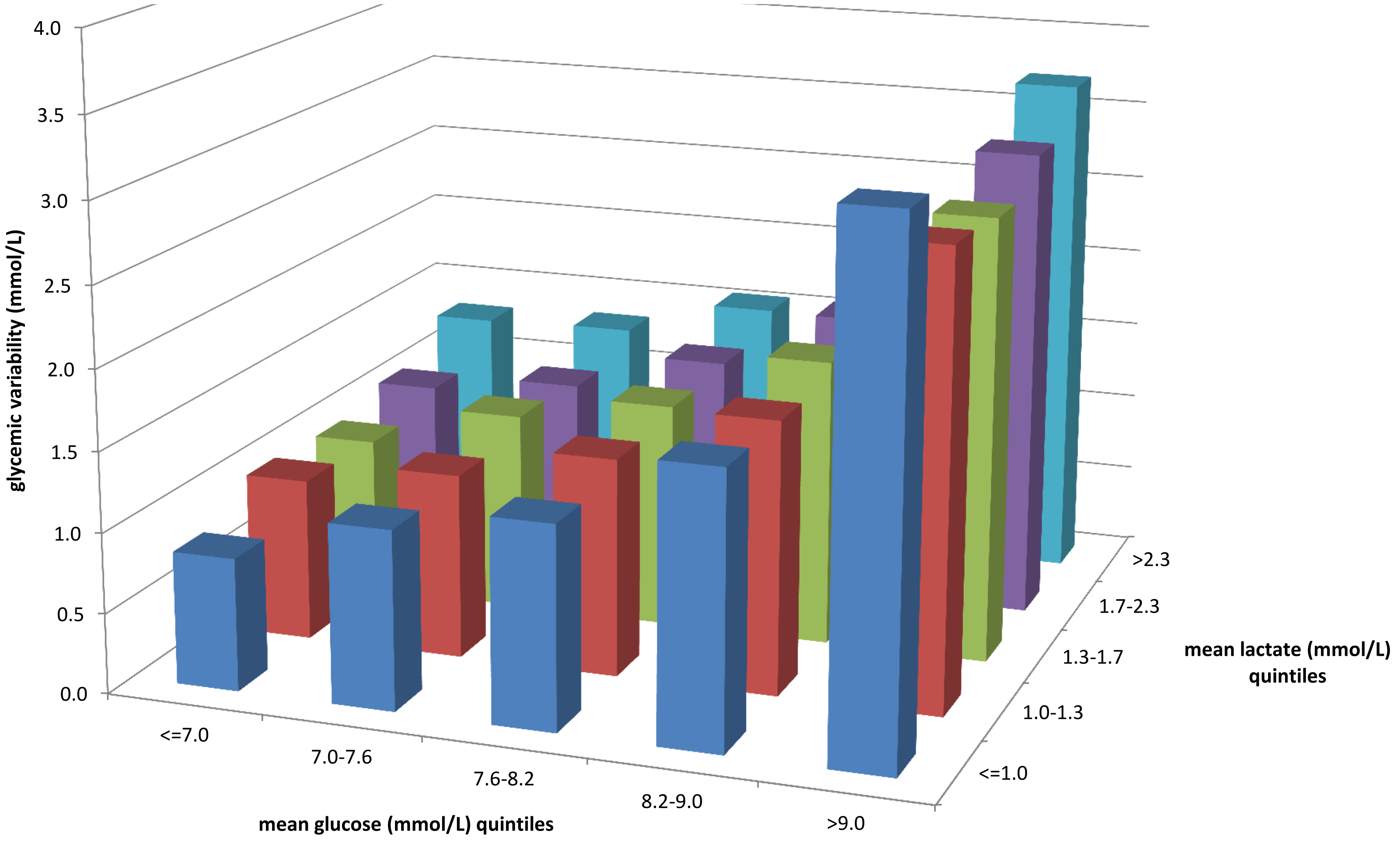

Legend for supplementary figure 15.

Glycemic variability for combined lactate and glucose quintiles.

Glycemic variability in mmol/L during the first 24 hours after ICU admission. Note that variability (i.e. the standard deviation) increases with the mean glucose level.

Suppl. figure 16 – Predicted mortality (%)

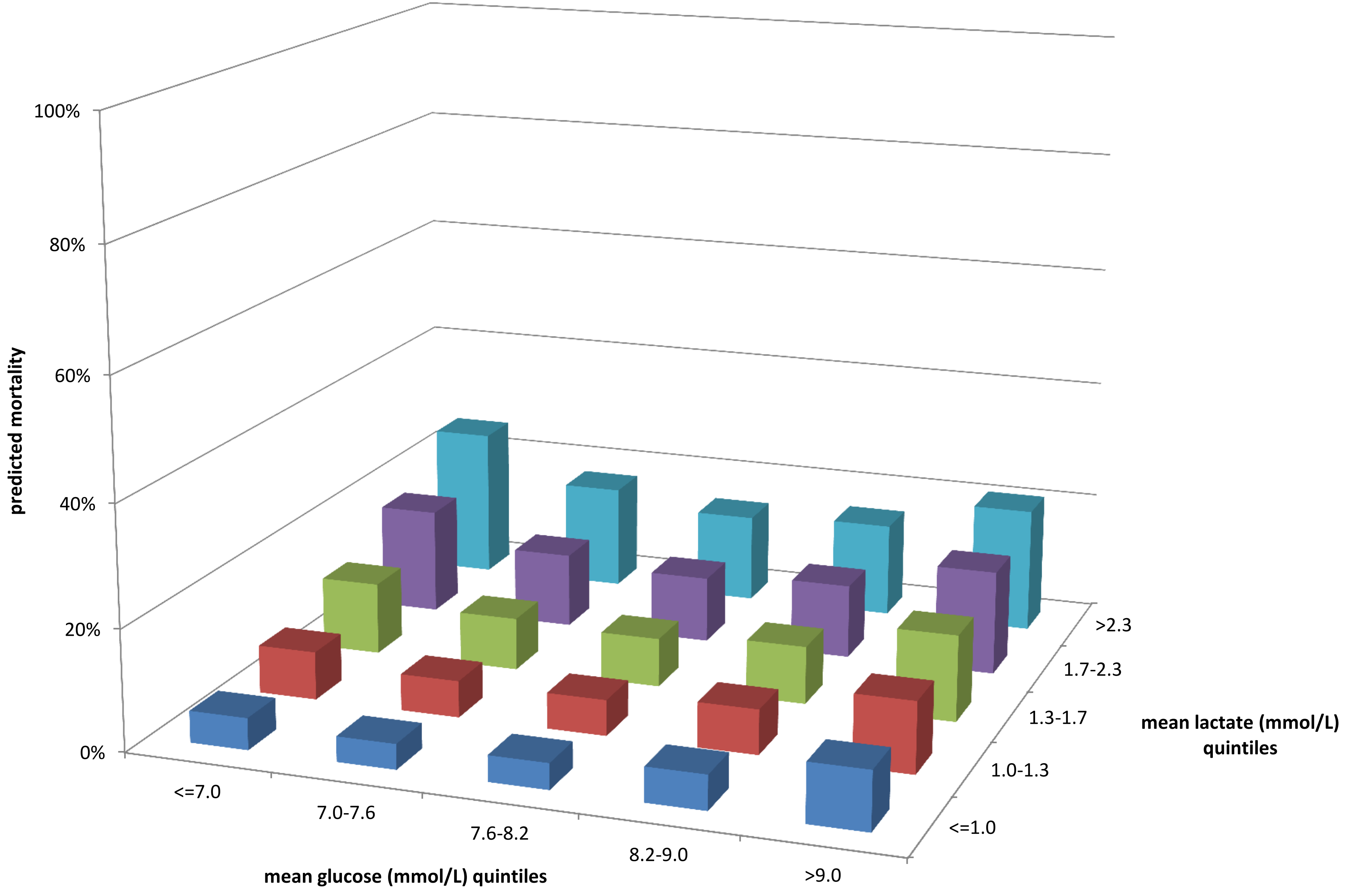

Legend for supplementary figure 16.

Predicted mortality calculated from the multivariate model with glycometabolic variables.

Predicted mortality for combined lactate and glucose quintile calculated from the multivariate logistic regression with only glycometabolic parameters (lactate quintiles, glucose quintiles, $(\text{glucose quintile-mean glucose quintile})^2$, and interaction term). This graph serves as comparison to the observed mortality for combined lactate and glucose quintiles shown in fig. 3 in the main manuscript.

End supplementary material
